# Assessment of Machine Learning Models to Identify Port Jackson Shark Behaviours Using Tri-Axial Accelerometers

**DOI:** 10.3390/s20247096

**Published:** 2020-12-11

**Authors:** Julianna P. Kadar, Monique A. Ladds, Joanna Day, Brianne Lyall, Culum Brown

**Affiliations:** 1 Department of Biological Sciences, Faculty of Science and Engineering, Macquarie University, Sydney, NSW 2109, Australia; culum.brown@mq.edu.au; 2 Marine Ecosystems Team, Wellington University, Wellington 6012, New Zealand; monique.ladds@gmail.com; 3 Taronga Institute of Science and Learning, Taronga Conservation Society Australia, Sydney, NSW 2088, Australia; jday@zoo.nsw.gov.au; 4 Royal (Dick) School of Veterinary Studies, The University of Edinburgh, Easter Bush Veterinary Centre, Midlothian EH25 9RG, UK; brianne.lyall@spca.nz

**Keywords:** machine learning, accelerometer, model selection, benthic, elasmobranch, epoch, support vector machine, foraging

## Abstract

Movement ecology has traditionally focused on the movements of animals over large time scales, but, with advancements in sensor technology, the focus can become increasingly fine scale. Accelerometers are commonly applied to quantify animal behaviours and can elucidate fine-scale (<2 s) behaviours. Machine learning methods are commonly applied to animal accelerometry data; however, they require the trial of multiple methods to find an ideal solution. We used tri-axial accelerometers (10 Hz) to quantify four behaviours in Port Jackson sharks (*Heterodontus portusjacksoni*): two fine-scale behaviours (<2 s)—(1) vertical swimming and (2) chewing as proxy for foraging, and two broad-scale behaviours (>2 s–mins)—(3) resting and (4) swimming. We used validated data to calculate 66 summary statistics from tri-axial accelerometry and assessed the most important features that allowed for differentiation between the behaviours. One and two second epoch testing sets were created consisting of 10 and 20 samples from each behaviour event, respectively. We developed eight machine learning models to assess their overall accuracy and behaviour-specific accuracy (one classification tree, five ensemble learners and two neural networks). The support vector machine model classified the four behaviours better when using the longer 2 s time epoch (*F*-measure 89%; macro-averaged *F*-measure: 90%). Here, we show that this support vector machine (SVM) model can reliably classify both fine- and broad-scale behaviours in Port Jackson sharks.

## 1. Introduction

Sensors have been applied widely to animals to understand the movements and behaviours of previously unobservable species or events. Advances in biologging have led to the quantification of behaviours of many species throughout the marine environment, extending our limits of observation. Acoustic and satellite telemetry, for example, have been widely applied to examine the large-scale movements of animals over long temporal scales (days–years) but do not have the capability to examine ecologically important fine-scale behaviour patterns (seconds–minutes) such as foraging [1,2]. Here, we define fine-scale movements as those that occur over very brief periods of time: less than two seconds, and broad-scale behaviours occurring over greater than 2 s to mins. Many of these fine-scale movements have applied significance yet are difficult to observe in wild marine animals without the use of animal-borne image recorders (e.g., foraging) [3]. Through identifying and quantifying fine-scale movements, we can increase our understanding of how each movement contributes to individual fitness as well as addressing broader questions such as examining collective species impacts on their surrounding ecosystems [4]. Accelerometers can address these knowledge gaps.

The development of increasingly efficient and inexpensive memory and battery capacity has enabled the miniaturization of biologging technology, including accelerometers, and made fine-scale (seconds–minutes) analysis possible [5]. Loggers are capable of detecting movements in three dimensions and recording this movement at hundreds of times per second (up to 800 Hz), providing unparalleled detail of animal movements [6]. These massive data sets offer novel challenges in terms of data analysis and interpretation.

Currently, many methods exist for analysis of accelerometry data which include various machine learning approaches that are used to categorise behaviours of interest. Supervised and unsupervised learning techniques offer automatic classification of behaviour categories, unsupervised learning through a set of defined rules that cluster unlabeled datasets [7] and supervised learning, through labelling or ground-truthing data [8]. Supervised learning requires training and can be biased because of this hands-on approach; yet, it has the potential to provide highly accurate and detailed results. These results can increase in accuracy if trained to identify fewer behaviour categories from relatively larger sample sizes, or longer epochs of time [9]. The feature characteristics used to describe each behaviour also have a high degree of influence on the overall performance of the model and are chosen carefully to reflect the biomechanics of the behaviours of interest [10]. Many methods also exist for applying different types of machine learning algorithms, each with their own advantages and disadvantages, that perform at different levels when classifying accelerometry from a given dataset [11,12,13]. Thus, accelerometer analysis techniques require the application of custom solutions and the trial of multiple algorithms to find the ideal candidates for optimal performance. A key issue is the difficulty in classifying fine scale behaviours (<2 s) in parallel with behaviours occurring at a relatively larger temporal scale (>2 s-mins).

Several studies have used machine learning model selection to optimise identification of focal behaviours, such as locomotion and foraging (e.g., [14]). In these studies, the focus has been on ensemble and non-probabilistic learning methods and fewer studies consider multiple categories of classical machine learning algorithms (e.g., logic-based, ensemble and neural networks) during the selection process. Additionally, recently developed state-of-the-art boosting algorithms such as XGBoost [15] that are currently widely applied in data mining are seldom taken advantage of in animal accelerometry studies. Leoni et al. (2020) built an XGBoost model for Olive baboon behaviours with an average accuracy for a single behaviour of 94.5%, a very high rate [16]. This outcome showcases the benefits boosting models may have over bagging models, such as the commonly used random forest. This advantage is likely especially true for datasets with imbalanced behaviour classes that are often found in animal accelerometry because of the short duration and rare occurrence of some behaviours [9]. The use of an optimal model will ensure balanced accuracy between behaviour classes that span varying temporal scales. In sharks, multiple categories of algorithms have been applied to juvenile lemon sharks and produced higher performance for fine- and broad-scale behaviours using a combined voting ensemble model [12]. Within benthic species of sharks, Karan et al. (2019) applied k-nearest neighbours models using various frequency transformation techniques to identify feeding encompassing prey capture, handling, chewing and headshaking in horn sharks (*Heterodontus francisci*) [17]. Feeding was identified in parallel with locomotion behaviours for horn sharks; however, at a cost to class-specific accuracy for feeding. Despite considerable advancements, there is room for further improvement by assessing multiple classical and state-of-the-art machine learning models for small benthic species of shark, which compose the majority of shark species.

In this study, we aimed to identify both fine-scale and relatively broader-scale Port Jackson shark behaviours with high accuracy by assessing the performance metrics of multiple machine learning models and epochs. The ethogram consisted of resting and swimming (broader temporal scale; >2 s-mins) and vertical swimming and chewing as a proxy for foraging (finer temporal scale; <2 s). Port Jackson sharks are a benthic, nocturnal, non-obligate ram-ventilating species that spend the majority of their time resting on the benthos [18]. They aggregate in large numbers during winter breeding season, displaying high site fidelity [19], and it is probable that they exert large effects on the surrounding kelp forests through regulating echinoderm species that consume kelp habitats [20,21]. Thus, identifying feeding behaviour is both important in the context of individual fitness, but also has broader ecological implications. Chewing, or mastication-like behaviour, within this species, is most often performed while resting. This context is ideal because it reduces the likelihood of other signatures in the accelerometry data (e.g., tailbeats) that may occur in synchrony with feeding masking the signature of this fine-scale behaviour. Resting and swimming represent periods of inactivity and activity, and sex, time of day and sex-specific seasonal activity have all been shown to influence activity in this species [18]. Accelerometers are a potential candidate for assessing the impacts of environmental variables upon activity and, in some cases, require validation to address these unknowns in free-ranging sharks. The final behaviour measured, vertical swimming, is a higher speed locomotion with presumably high energy costs, occurring almost exclusively in captivity against vertical surfaces of enclosures. Thus, the aims of this study were to (1) determine which epoch length (1 s: 10 samples; or 2 s: 20 samples) used while summarising the data led to better model performance, and (2) compare and contrast the effectiveness of eight different candidate machine learning models including logic-based, ensemble, and neural network algorithms.

## 2. Materials and Methods

### 2.1. Captive Observations

Captive experiments were conducted with four adult female Port Jackson sharks (Table 1) at Taronga Zoo in Sydney, Australia (−33°50′ N, 151°14′ E), in October 2015. Sharks were captured from Quarantine Point (−33°82′ S, 151°28′ E) or Fairlight Beach (−33°48′ S, 151°16′ E), Sydney Harbour and transported to an open-roof enclosure at Taronga Zoo. All individuals were measured for total length and weighed upon arrival. The enclosure measured 17.8 × 8 m with a maximum depth of 3.3 m and received constant water flow sourced from Sydney Harbour that was mechanically filtered.

Temperature and water quality within the enclosure were measured daily but reflected ambient conditions. The group of sharks was fed daily at 15:00 with soft and hard-shelled prey typically found in their diet. To replicate caves that the sharks are found naturally inhabiting, three hides were placed on the floor of the enclosure. Sharks acclimated to the enclosure for 48 h prior to experiments.

This study was completed under permits from Macquarie University Animal Ethics Committee (ARA-2014/015) and Taronga Conservation Society Australia Animal Ethics Committee (3a/06/15). Wild sharks were collected and released under NSW DPI Fisheries Scientific Collection Permit P08/0010-4.2.

### 2.2. Accelerometer Attachment and Specifications

Accelerometers were attached to the first dorsal fin at two points to prevent instability and shifting as well as damage to the skin of the individuals. Two stainless steel 14-gauge Sureflo IV surgical catheters (Terumo, Tokyo, Japan) were used to puncture the anterior end of the first dorsal spine. Suture thread, Prolene size 1 (Ethicon, Somerville, NJ, USA), was then threaded through the catheters and used to secure the accelerometer against the skin.

**Figure 1:**
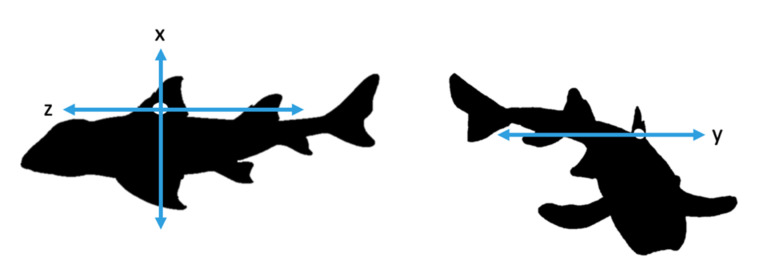
Representation of x, y and z axes of accelerometer attached to the Port Jackson sharks.

During experimentation, two sharks were tagged with accelerometers at a time and allowed to swim freely in the enclosure. We used tri-axial accelerometers (Cefas G6a+: 40 mm × 28 mm × 16.3 mm and weigh 18 g in air and 4.3 g in seawater, CEFAS technology Ltd., Lowestoft, UK) to measure the movement of the sharks on three axes: x, y and z (Figure 1). Accelerometers were set to record at +2 g and 10 samples per second (10 Hz). Higher frequency recording was avoided to increase memory efficiency in the tags. This facilitated the aim that this relatively low sampling frequency would lead to longer duration deployments in the wild, though were still capable of detecting the behaviours of interest [22]. Data were recorded at random blocks of time during daylight hours with a minimum recording time of two hours and a maximum of six hours. The accelerometers remained on the animal for five days.

### 2.3. Video Observations

Video cameras (GoPro Hero4, San Mateo, CA, USA) were submerged in three locations in the enclosure to record movements of the tagged sharks during daylight hours for five days. Videos from the three cameras were imported into Adobe Premier Pro CC (Adobe Systems Inc., San Jose, CA, USA) where they were synced so they could be viewed at the same time in a single movie file. Videos were scored manually using Excel (Microsoft Corp., Redmond, WA, USA) and QuickTime Player (Apple Computer Inc., Cupertino, CA, USA) according to an ethogram (Supplementary Table S1) developed from the video footage of the unique behaviours: resting, swimming (in the water column or on the floor), vertical swimming, and chewing on prey. The videos were coded continuously; however, multiple diverse behaviours that were originally recorded including foraging, burst swim, chaffing, roll and head shake were later labelled as ‘other’ because of a lack of occurrences. The models were not trained on the ‘other’ behaviour category due to the inconsistency within it and the difficulty this presents for the repetitive training process [13]. Periodically, the sharks swam out of view of the recording; as a result, those sections were labelled as ‘out of camera’ within the behaviour coding and excluded from the final dataset. A subset (30%) of the behaviour coding was completed by two different coders and compared for validation. Over 97% of coding resulted in the same coded behaviour; therefore, the remainder was completed by one coder. Following behaviour coding, videos and acceleration output were synchronised to create annotated datasets. To synchronise video footage labels to accelerometry data, the accelerometer was hit against a hard surface during filming to create a spike in the data to ensure an exact match between the accelerometer and video footage as per Ladds et al. (2016) [9].

### 2.4. Data Processing and Feature Analysis

After the removal of the accelerometers from the sharks, data from trials were downloaded using G5 Host software (version 6.7.0, CEFAS technology Ltd., Lowestoft, UK). To account for and avoid abnormal behaviour resulting from catching and handling effects, the first 24 h of data were excluded from analysis.

Summary statistics for the machine learning models were created from 1 and 2 s epoch lengths to classify each of the four behaviour classes. Epochs are fixed time segments (sliding sample windows) that help reduce noise while also reducing complexity in the dataset. These epoch lengths were chosen due to the fact that two behaviours (vertical swimming and chewing) took place over a fine temporal scale (i.e., <2 s). The epoch lengths were considered short enough to capture a single behaviour but long enough to allow for differentiation between the behaviour classes [23]. The 1 and 2 s epochs were composed of 10 and 20 data points, respectively, (Supplementary Table S2). Each summary statistic from the epochs contained accelerometer data from one behaviour only. Static and dynamic acceleration were calculated from the raw acceleration data. To obtain static acceleration, raw axes were smoothed by applying a moving average across five samples due to the relatively small body size of the sharks and the partial focus on the classification of finer-scale behaviours. Dynamic acceleration was calculated by subtracting static acceleration from raw acceleration [24]. The six characteristics calculated from the data were the mean, standard deviation (SD), minimum, maximum, skewness, and kurtosis for the features: *x*-axis, *y*-axis, *z*-axis, overall dynamic body acceleration (ODBA), vectorial dynamic body acceleration (VeDBA), movement variation, energy, pitch, and roll (see Table 2 for equations). In addition, 10th (Q10) and 90th (Q90) percentiles were calculated for ODBA, VeDBA, movement variation, energy, pitch and roll. This resulted in 66 summary statistics to potentially describe each behaviour event.

### 2.5. Swim Column and Swim Floor Behaviours

Due to the similar biomechanics between swimming on the floor and swimming in the water column, the two behaviours were compared with parametric tests on a range of summary statistics to evaluate whether they should be combined into a general ‘swimming’ category. The comparison features were: SD of ODBA and VeDBA, mean of ODBA, and maximum of VeDBA which were compared using two sample t-tests for mean differences. If the behaviours were not significantly different (*p* < 0.05), they would be combined into the general ‘swimming’ category for the modelling analysis.

### 2.6. Classification Models

Three types of classification models were applied to this dataset: logic-based, ensemble methods and neural networks (Table 3). Within these categories, eight candidate models were compared for performance that had previously been shown to perform well for classifying ethograms developed from accelerometers (e.g., [6,9,11,12,13,25]). All classification models were developed in R version 4.0.3 [26].

For each model, behaviour events were split into a train (evaluation) and test (validation) sets using 80% and 20% of the data, respectively. Repeated k-fold cross-validation was used to tune model hyperparameters using 10-fold cross-validation with five repeats. For subsampling, we used the function upSample in the caret package to randomly up-sample the minority classes in the training set (with replacement) to be the same size as the majority classes to avoid model bias during testing [33].

### 2.7. Model Performance Assessment

Cohen’s kappa values were compared between the epochs to assess the influence of random chance upon overall accuracy [34]. Results of the models were reported as cross-validation scores and out-of-sample scores, which included accuracy, sensitivity (recall), specificity, precision, *F*-measure, and macro-averaged *F*-measure (Table 4). Accuracy was calculated as a simple ratio between the amount of correctly classified events to the total amount of events. This is a good indicator for overall accuracy; however, the remaining performance metrics were used to assess class-specific accuracy, which is essential for considering the classification success of rare behaviour events [12]. Sensitivity was used to determine the percentage of events in a class that were correctly identified, and specificity showed the percentage each model correctly identified as not belonging to the class. Class *F*-measure indicated the balanced mean between precision and sensitivity, while macro-averaged *F*-measure corrected for the lack of occurrence of the relatively finer-scale behaviours by giving equal weight to all categories.

All performance assessments were completed using the caret package [33] in R version 4.0.3 [26].

## 3. Results

### 3.1. Sampling

All acceleration data loggers remained attached to individuals for five days before they were manually removed from the dorsal fin of each shark (*n* = 4). The footage equated to 4183 and 8215 captured data points for behaviour events for the 1 and 2 s epochs, respectively (Supplementary Table S2). Chewing and vertical swimming behaviours contained the least number of examples owing to their rare, temporally fine-scale occurrence and difficulty to capture on video footage. Swimming had the greatest number of examples, followed by resting, chewing and finally vertical swimming (Supplementary Table S2).

### 3.2. ODBA for All Behaviours

**Figure 2:**
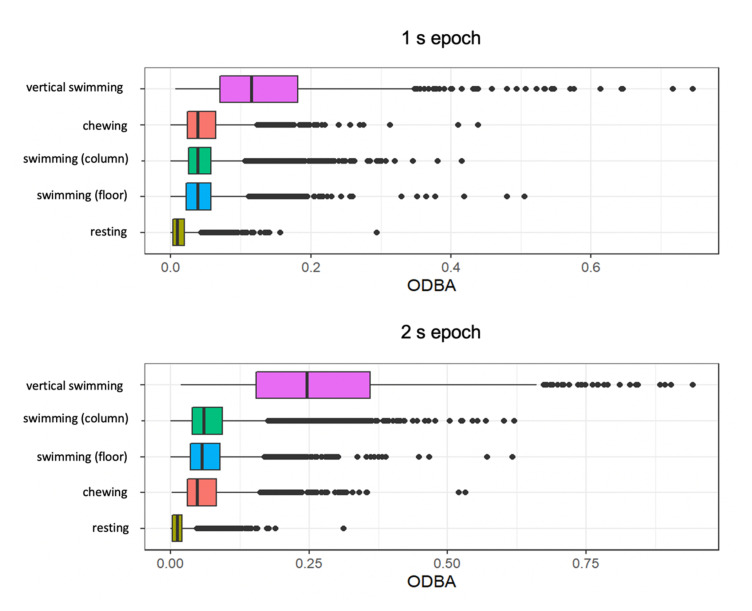
Boxplots of overall dynamic body acceleration (ODBA) for initial ethogram of behaviours. Note, swimming in the water column and swimming on the floor were combined for subsequent modelling analysis.

Mean ODBA values were higher for the longer 2 s epoch of time. ODBA was comparable for swimming in the water column and swimming on the floor (Supplementary Figure S1). Vertical swimming showed higher ODBA values than all behaviours. Resting showed the lowest variation in mean values and lowest values overall (Figure 2). Chewing behaviour had higher mean ODBA in the 1 s epoch than swimming behaviours and lower ODBA than swimming behaviours in the 2 s epoch (Figure 2).

### 3.3. Swimming in the Water Column vs. Swimming on the Floor

Mean values and ranges of the summary features including mean ODBA, the SD of ODBA, the SD of VeDBA, and maximum VeDBA did not differ significantly for swimming on the floor and in the water column (Supplementary Figure S1, Table S3). The metrics were similar for both behaviours, and two sample t-tests showed no significant difference between swimming in the water column and swimming on the floor for mean ODBA (*p* = 0.565), the SD of ODBA (*p* = 0.689), the SD of VeDBA (*p* = 0.576) and maximum VeDBA (*p* = 0.70) (Supplementary Figure S1, Table S3). Thus, the two groups were combined into the ‘swimming’ behaviour class.

### 3.4. Description of Behaviours

**Figure 3:**
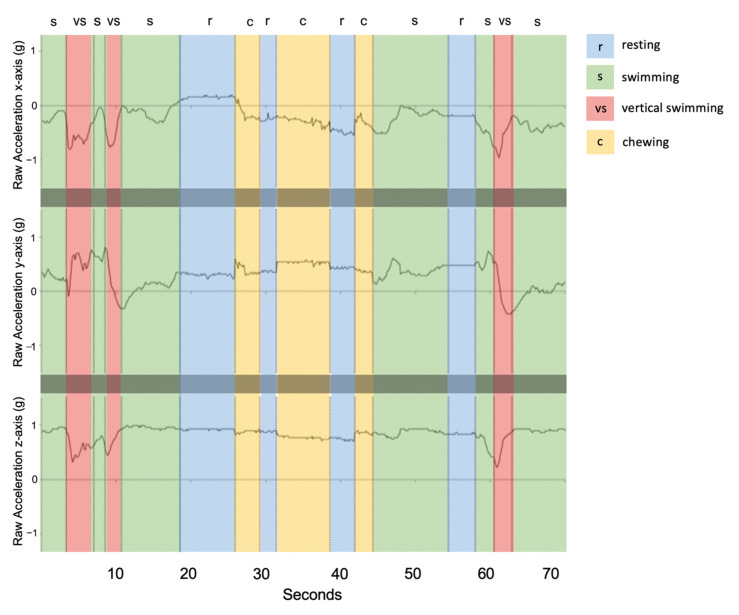
Example of raw acceleration data over a 70-s time window (shark ID 5541) engaging in each of the four behaviour classes. Vertical dashed lines indicate transitions between behavioural states where r: resting (blue), s: swimming (green), vs: vertical swimming (red), and c: chewing (yellow).

Resting behaviour displayed little to no change across the three axes (Figure 3) and exhibited the lowest acceleration values. Infrequently, resting events showed small peaks in cases of jostling disturbance from group resting behaviour. Chewing behaviour took place over short durations, (<2 s) and was most distinctly visible over the x and y-axes (Figure 3). Compared to resting, the *x*-axis for chewing was further below the *y*-axis and exhibited small, quick rhythmic peaks for each mastication movement (Figure 3). Swimming events were most variable on the *y*-axis with an increase in magnitude during the undulation of the body from left to right (Figure 3). The clearest 3-axis movement and highest variation in g values occurred for vertical swimming behaviour, during which the tailbeats were more pronounced, and the body was propelled in a vertical direction (Figure 3).

### 3.5. Model Classification

**Figure 4:**
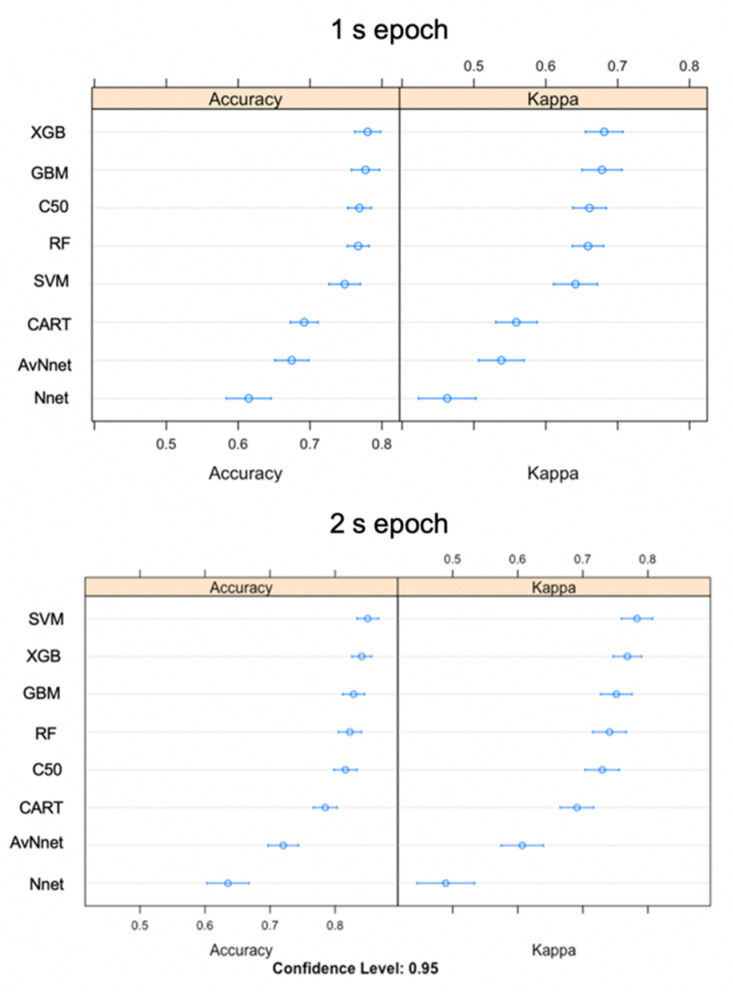
Accuracy and kappa values displaying model fit for eight classification models built using either 1 s or 2 s epoch. Higher values are a better fit.

For the training datasets using the 1 s epoch, the three highest performing models according to overall accuracy (and Cohen’s Kappa) were eXtreme gradient boosting (XGB) (78%), stochastic gradient boosting (GBM) (77.7%), and C50 (76.9%) (Figure 4). Training accuracy for the 2 s epoch was higher (support vector machine (SVM) (85%), XGB (84.1%) and GBM (82.8%)) (Figure 4). The models with the highest accuracy also had the highest kappa, so they performed well compared to the control of a random classifier while taking uneven classes into account. Overall, the mean training performance of the top three 2 s models was 6.5% higher than 1 s epoch models (Figure 4). Neural networks were the two lowest performing models.

Training accuracy was high across all ensemble learning models and relatively lower for both neural networks. Each algorithm performed better on the training data set than the testing set except in the case of Nnet 1 and 2 s epochs and AvNnet 2 s epoch results. The three models with overall highest accuracies were from the 2 s epoch; SVM (89%), RF (89%), and XGB (87.8%) (Table 5). The best model parameters for all models are listed in Supplementary Table S4.

**Figure 5:**
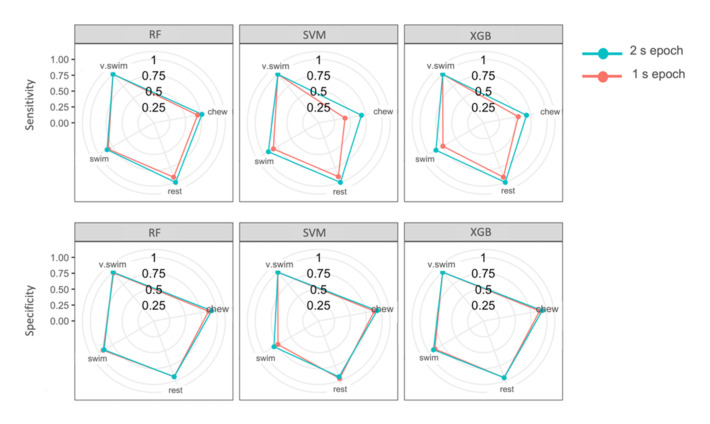
Sensitivity and specificity of random forest (RF), support vector machine (SVM) and eXtreme gradient boosting (XGB) of the four behaviour classes for the 1 and 2 s epochs. The centre of the circles is 0% sensitivity or specificity and the performance increases moving outward.

The top three models (SVM, RF and XGB) all identified behaviours with high overall accuracy, high sensitivity and specificity for each behaviour class. Chewing was most often misclassified as swimming for all three models. Resting was most often misclassified as swimming behaviour. Vertical swimming, a very dynamic behaviour, was not misclassified in the top three performing models (Table 6). Sensitivity of all behaviours except vertical swimming improved from the 1 s to the 2 s epoch models (Table 6; Figure 5). Specificity remained similar throughout both epochs, though the swimming category in SVM improved markedly by 8.2% from 1 s to 2 s epochs (Table 6; Figure 5). Sensitivity for the chewing category with SVM improved most notably by 26.3% when comparing 2 and 1 s epochs (Table 6; Figure 5).

### 3.6. Feature Importance

**Figure 6:**
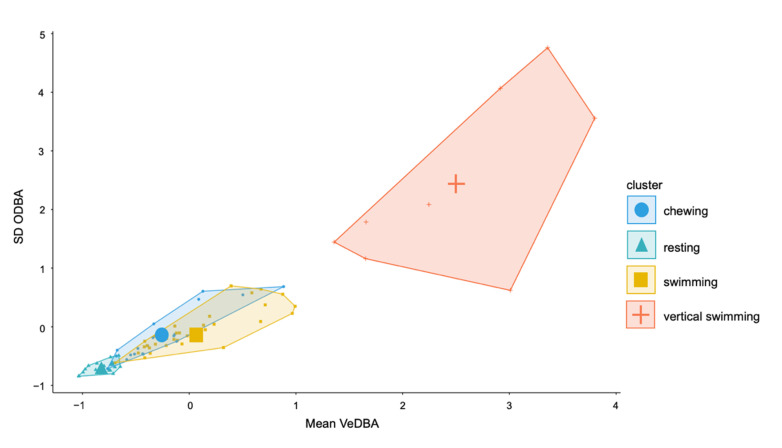
Cluster plot of SD ODBA and mean vectorial dynamic body acceleration (VeDBA) for the 2 s epoch for four behaviours.

Variable importance for the top performing models was similar across the top three performing 2 s models. VeDBA Q90 and ODBA Q90 were the most important features followed by mean VeDBA and maximum VeDBA (Supplementary Figure S2). Q90, SD and maximum values were the most valuable characteristics to describe each behaviour event (Supplementary Figure S2). Mean VeDBA and ODBA SD were particularly important for the highest performing SVM model (Figure 6).

## 4. Discussion

Accelerometers have been used to classify a wide variety of behaviours achieving varied performance depending on the duration of focal behaviours [35], the number of ethogram categories [36], and the chosen machine learning method [13]. In some cases, higher performance is accomplished for behaviours occurring over a longer temporal scale (e.g., [37,38]). With adult Port Jackson sharks we showed that fine-scale behaviours (those occurring in less than 2 s) can be classified with good performance (>78% *F*-measure) in parallel with broader-scale behaviours (those occurring over time scales greater than 2 s). In this study, we trained machine learning models to categorise and identify four behaviours: resting, swimming, vertical swimming and chewing. Different epoch lengths and model types were tested, and we found that changing both of these factors contributed to variation in prediction performance. By selecting four behaviours to construct the ethogram and optimising the models for fine-scale behaviour identification, we ensured reliable identification of key behaviours representing foraging (chewing), migration and residency, and activity (resting, swimming and vertical swimming).

### 4.1. Machine Learning

Classification using supervised machine learning has frequently been used with accelerometry datasets from animals. Multiple algorithms are available to solve classification problems, and examining diverse methods has been recommended to select the most appropriate option [39]. For this species and the given ethogram, the classical machine learning model that classified with the best overall and behaviour-specific performance was SVM with a radial basis function kernel (accuracy: 89%; macro-averaged *F*-measure: 90%). SVM has previously performed well with animal data sets in penguins [40], dairy cows [35], fur seals and sea lions [9] and a variety of other species including alligators [41]. These models function by portioning a feature space into two or more groups. In simple cases, the boundaries they create are linear resulting in groups that are separated by planes. In non-linear cases, as used here, SVM can use the kernel trick to divide data using hyperplanes in higher dimensional spaces to create boundaries. After division, the two closest events to the hyperplanes are known as support vectors. These optimal boundaries are then used to predict classes for a new set of observations. This likely worked with the current ethogram because, by expanding the dataset, the SVM was able to compute an optimal hyperplane, creating distinct boundaries between swimming, resting and chewing behaviour.

Random Forest had the same overall accuracy of 89% as SVM; however, *F*-measure of chewing was 4.7% lower than the SVM (Table 6). Neural network performed moderately well but with the lowest accuracy overall for both epochs and model types (Table 5). In a study on juvenile lemon sharks (*Negaprion brevirostris*), a voting ensemble model combined logistic regression, artificial neural networks, two random forests and gradient tree boosting models to achieve a macro-averaged *F*-measure of 88%. The voting ensemble model achieved high class-specific *F*-measure for fine-scale behaviours headshake (79.1%) and burst swim (73.7%) [12]. In this study, we were able to obtain similar levels of performance without using a voting ensemble model, simplifying the classification process. Lemon sharks continue swimming during foraging events leading to behavioural synchrony. This may cause greater difficulty in categorizing headshake behaviour, similar to the issue of categorizing short-lived foraging behaviour in baboons while they are travelling [42]. In contrast, Port Jackson sharks perform chewing behaviour in isolation which is likely the reason for the similar model performance accomplished to other sharks using classical machine learning methods alone and a 10 Hz sampling frequency [22]. This study had comparable metric performance for both SVM and RF models with the difference that SVM is more suitable for fine-scale behaviours and RF may be more readily applied to a binary ethogram consisting of resting and active (broad-scale) behaviour (Table 6). Detection of four mutually exclusive behaviours in sheep by Fogarty et al. (2020) was best classified using SVM (76.9%), while activity and posture from the same dataset were most accurately detected by CART and linear discriminant analysis, respectively [43]. This suggests the behaviours of interest will dictate model choice. However, it may be more efficient to design models with this adaptability to levels of behavioural resolution in mind. This will ensure that presently developed models can be adapted to future studies on the same species [10].

### 4.2. Ethogram

Five behaviours were classified in total, two were combined because they were largely indistinguishable (swimming on the floor and swimming in the water column) and others occurred too rarely (<10 times) to include in modelling analysis. Resting and swimming occurred most often, while the finer-scale behaviours—chewing and vertical swimming—occurred with less frequency. These behaviours represent important parts of the Port Jackson shark activity budget that both captive and wild sharks display. Vertical swimming is rarely observed in the wild, but in captivity sharks frequently come into contact with the walls of the enclosures. Future outcomes from correctly classified baseline behavioural data with robust sample sizes can directly contribute to knowledge of individual and population dynamics and inform management and welfare of shark species, many of which are threatened and vulnerable to anthropogenic impacts [44,45]. Quantifying swimming and resting alongside foraging behaviours in wild sharks would allow researchers to gain insights into Port Jackson shark time-activity budgets and energetics to better understand their ecology [46].

Within the modelling, SVM misclassified resting as chewing and swimming equally as often, the other top two performing models mistook resting as swimming most of the time, regardless of epoch. Though these behaviours are visually distinctive, shorter epoch lengths may have represented immobile behaviours with higher than typical dynamic movement values and mobile behaviours with lower dynamic movement values. Summary features which may be responsible for this are relatively higher or lower ODBA and VeDBA values resulting from a short window of time that has summarised a periodic acceleration peak or trough within the behaviour event.

Swimming was most often misclassified as chewing and chewing behaviour as swimming across all candidate models. Chewing behaviour is representative of a successful foraging attempt and quantified mastication movements as the individuals consumed a diversity of prey, including hard-shelled organisms. The accelerometer placement on the dorsal fin meant that all behaviours could be reliably recorded including swimming; however, this created a subtle signature with small peaks on the *x*-axis for chewing. Like chewing, vertical swimming occurs for short periods of time; however, it resulted in a high ODBA value, which also quantified its costly nature (Figure 2). Though this behaviour occurred least frequently of the four categories, it was identified with high performance (class *F*-measure: >93%) for all epochs and models, excluding neural networks. Unsurprisingly, this result was possible because the acceleration axes shifted by ~90° during this dynamic motion that constituted increased tailbeats and high ODBA values. Increased ODBA results in increased muscle contractions and, therefore, more oxygen usage [47,48,49,50,51]. In captivity, individuals may be displaying persistent swimming and bouts of vertical swimming behaviour for increasing lengths of time during the weeks when migration usually occurs [18]. This demonstrates that vertical swimming likely has significance for individual welfare in captivity due to increased activity. Quantifying this behaviour in captive environments and addressing its persistence may contribute to individual health and welfare of migratory sharks held in zoos and aquariums.

Four selected categories were a low enough number to achieve high model performance for all behaviours. This abridged ethogram did not encompass all ecologically important behaviours of Port Jackson sharks; however, it can potentially be applied to foraging, migration and resident breeding season [19]. Chewing behaviour was quantified using a diversity of deceased prey including squid, clams and urchins; however, bony fish and other live prey were not included in chewing behaviour validation. So, further variability in this behaviour may exist from larger or live prey items. Resting and swimming behaviours may also differ in captivity, affecting model performance. Klay et al., 1977, hypothesised that limited size in aquariums requires increased turning rates and a captive study on sand tiger sharks (*Carcharias taurus*) found reduced gliding behaviour for some individuals and the presence of asymmetrical swimming (clockwise or counter-clockwise) for the majority of the time [52,53]. In a captive environment, swimming behaviour signatures may show differences in posture and acceleration because of continual turning due to short lengths of aquaria or lab enclosures. Model performance may also be affected by wild resting behaviour occurring in turbulent water, which may result in low peaks from surging waves [54]. Additionally, behaviours including gliding, burst swimming, rolling and foraging (pre-prey capture) were recorded for too few instances to train a robust model for tagged individuals. The models developed here considered the behaviours of four individual sharks within a captive setting to allow for the final model to be applied to other individuals; however, it is possible that further individual variation will be present in wild populations and how successful the model is in the wild context may depend on this [55]. Indeed, the models may require further validation in the wild perhaps in combination with footage from underwater drones.

If this final model is applied to wild sharks, the SVM will classify all shark movements as one of the four behaviour categories and will over-represent their prevalence because sharks perform a range of behaviours that are not represented by the models. During initial video analysis, an ‘other’ category was labelled in the validation of accelerometer data but was not used to train the model because the behaviours within this category were too diverse to reliably train a model [13]. Rather than constructing an activity budget for wild sharks that we cannot observe directly, this model has the potential to predict the presence, absence or rate of chewing behaviour per set blocks of time (e.g., per hour; [12]).

### 4.3. Epochs

Changing the epoch from 1 s to 2 s improved sensitivity and specificity for all behaviour classes in the top three performing models (Table 6). Having the option of two relatively short epochs allowed for fine-scale behaviours to be distinguished consistently. The 2 s epoch improved chewing identification for the final SVM model by 26.3% (sensitivity) and 7.9% (specificity). All models were able to identify vertical swimming consistently across both epochs due to its highly distinguishable features. Overall, 2 s epochs improved sensitivity to a greater degree than specificity for these categories. In other words, more samples ensured that a great number of chewing behaviour events were correctly identified as chewing; however, this change in epoch did not improve instances where resting and swimming behaviour were correctly identified as not chewing.

It is important to point out that, by choosing these relatively-short-duration epochs, some models were compromised to a small degree in their ability to identify broader-scale behaviours: resting and swimming. This is because repetitive behaviours are more easily identifiable by models because of patterns in the data [35]. For example, swimming displays peaks for each tailbeat on the *y*-axis. Swimming was misclassified as chewing most often, possibly because the epoch did not allow for pattern recognition of periodicity within the tail-beat movement [54,56,57]. Despite this, swimming categorisation still maintained high performance metrics (91.4% sensitivity; 91.5% specificity). Longer epochs may enable better identification of rhythmic swimming movements.

### 4.4. Summary Feature Importance

The relative importance of summary features remained consistent across most models for the 2 and 1 s epochs. VeDBA Q90 was the most important summary feature across all models, and ODBA Q90 closely followed in 2 s epoch models (Supplementary Figure S2). ODBA Q90, however, was not an important variable for 1 s epochs. This may occur because higher ODBA values were mostly able to present in the relatively longer epoch, and the highest ODBA behaviour, vertical swimming, had lower ODBA values for the 1 s epoch than the 2 s epoch (Figure 2), resulting in closer overall ODBA values for the four behaviours. Other important features were VeDBA mean and maximum, and ODBA SD and mean. The models generally placed less importance on minimum, Q10, and features summarising x, y and z axes. Pitch was also a more important feature for the 1 s epoch, highlighting that by increasing the time epoch we create less reliance on posture than dynamic movements (Supplementary Figure S2). Identifying important summary features for this species could lead to compression of data onboard the tag, optimising energy and storage efficiency [58].

## 5. Conclusions

As accelerometers continue to be applied and as the technology improves, we stand to gain ever larger datasets that will require the advancement of machine learning techniques. These methods specify the importance of model selection in combination with epoch selection for reliable identification of behaviour categories. The SVM model using 2 s epoch summary statistics had the same overall accuracy as the RF model, however, macro-averaged *F*-measure showed better balance of identification between the classes, including fine-scale behaviours (<2 s). This selection process ensured the most reliable model was chosen and provided insight into the biomechanics behind Port Jackson shark behaviours, including very-short-duration behaviour events.

We have outlined our selection process in this study by considering the multiple options that are available for recording frequency, epoch length and algorithm type. We confirm that by placing the accelerometer near the center of mass of the animal (i.e., dorsal fin), defining of broad-scale (>2 s-mins) and fine-scale (<2 s) behaviours in parallel is possible. For the identification of feeding events alone, placement is likely best nearer to the head or on the mandible. Extending the epoch length is best for the identification of broad-scale behaviours, and the RF model may be most useful for these types of research questions. To understand fine-scale behaviours, a lower epoch length is beneficial for elucidating fine-scale behaviours using SVM models. Our study demonstrates that an SVM classical machine learning algorithm can be applied to tri-axial accelerometer data to build a reliable model for Port Jackson shark behaviour classification. This model has the potential to be applied to either captive or wild Port Jackson sharks to gain insight into welfare status and foraging ecology for this species. Through its application, this model can reduce pitfalls of classical observation techniques such as human observation time, error and observer effects upon shark behaviour.

## Figures and Tables

**Table 1:** Individual and experiment details for four sharks within the study.

ID	Sex	Total Length (cm)	Location of Capture
5541	F	127	Quarantine Point
5542	F	124	Quarantine Point
5544	F	117	Fairlight Beach
5545	F	120	Fairlight Beach

**Table 2:** Equations for six features used to calculate summary statistics for behaviour events.

Feature	Equation
ODBA	$=\left|X_{\mathrm{dyn}}\right|+\left|Y_{\mathrm{dyn}}\right|+\left|Z_{\mathrm{dyn}}\right|$
VeDBA	$=\sqrt{X_{\mathrm{dyn}}^{2}+Y_{\mathrm{dyn}}^{2}+Z_{\mathrm{dyn}\;}^{2}\;}$
Movement variation	$=\left|X_{i+1}-X_{i}\right|+\left|Y_{i+1}-Y_{i}\right|+\left|Z_{i+1}-Z_{i}\right|$
Energy	$={\left(X_{i}^{2}+Y_{i}^{2}+Z_{i}^{2}\right)}^{2}$
Pitch	$=\tan^{-1}\left(-\frac{X_{i}}{\sqrt{Y_{i}^{2}+Z_{i}^{2}}}\right)\times 180/\pi$
Roll	$=\mathrm{atan}2(Y_{I},Z_{i})\times 180/\pi$

**Table 3:** Model category, type, R packages used and short descriptions of classification models.

Model Category	Model Type	R Package	Model Description
Logic-based	Classification and regression tree (CART)	rpart [27]	- Lightweight and fast decision tree structure that allows for visibility of decisions. - However, they lack the complexity of other methods and may not perform as well as ensemble algorithms.
Ensemble	Bagging		
	Random forest (RF)	randomForest [28]	- Builds an ensemble of many independent decision trees using different sets of training data that are generated at random and replaced at each selection (known as bagging). - This large number of trees is used to create a consensus and results in the selection of the most common output that will lead to the maximum number of a class in a single node.
	Boosting		
	Support vector machine (SVM), with radial basis function	e1071 [29]	- Boosting methods fit trees on a modified version of the original data. - By training multiple models additively and in a sequence, these algorithms can identify the errors of weaker, single decision trees. - For example, GBM differs from RF in the order the decision trees are built and the method by which the results are combined. - SVM is an effective tool in datasets with large dimensionality (i.e., a large number of features).
	eXtreme gradient boosting (XGB)	xgboost [15]	
	C5.0 (C50)	C50 [30]	
	Stochastic gradient boosting (GBM)	gbm [31]	
Neural network	Feed-forward neural network (Nnet)	nnet [32]	- Influenced by the function and structure of biological neural networks and can learn highly complex patterns. - By using hidden layers, they create intermediary representations of data that other models cannot reproduce. - AvNnet fits multiple Nnet models and uses the average of the predictions from each constituent model.
	Model averaged neural network (AvNnet)	avnnet [33]	

**Table 4:** Performance metric equations used in model assessment. The index *i* is the behaviour event, *TP* = true positive, *TN* = true negative, *FP* = false positive, and *FN* = false negative. *M* is the number of behaviour classes.

Performance Metric	Equation
sensitivity	$=\frac{TP_{i\;}}{\left(TP_{i\;}+FN_{i\;}\right)}$
specificity	$=\frac{TN_{i}}{FP_{i\;}+TN_{i\;}}$
precision	$=\frac{TP_{i\;}}{\left(TP_{i\;}+FP_{i\;}\right)}$
*F*-measure	$=\frac{TP_{i\;}}{TP_{i\;}+\frac{1}{2}\left(FP_{i\;}+FN_{i\;}\right)}$
Macro-averaged *F*-measure	$=\frac{\sum_{i=1}^{M}F_{i\;}}{M}$

**Table 5:** Average (out-of-sample) test results of classification models for 1 and 2 s epochs.

Model	Test Accuracy	Macro-Averaged *F*-measure
2 s epoch		
SVM	89%	90%
RF	89%	89.2%
XGB	87.8%	88.6%
GBM	86.6%	87.2%
C50	84.1%	84.6%
CART	79.3%	81.7%
Nnet	75.6%	74.8%
AvNnet	75.6%	73.2%
1 s epoch		
SVM	76.8%	78.2%
RF	85.4%	84%
XGB	79.3%	78.1%
GBM	81.7%	81.5%
C50	79.3%	76.4%
CART	72%	73.3%
Nnet	73.2%	72.6%
AvNnet	70.7%	70%

**Table 6:** Confusion matrix for the test set of the summarised data. Values in bold are correctly classified behaviour events. V. Swim is vertical swimming behaviour.

	Predicted Behaviour				Performance Metric			
Observed Behaviour	Chew	Rest	Swim	V. Swim	Sensitivity	Specificity	Precision	*F-*Measure
SVM (2 s epoch test)								
Chew	**13**	0	1	0	68.4%	98.4%	92.9%	78.8%
Rest	2	**20**	2	0	100%	93.6%	83.3%	90.9%
Swim	4	0	**32**	0	91.4%	91.5%	88.9%	90.1%
V. Swim	0	0	0	**8**	100%	100%	100%	100%
SVM (1 s epoch test)								
Chew	**8**	2	4	0	42.1%	90.5%	57.1%	48.5%
Rest	3	**19**	2	0	90.5%	91.8%	79.2%	84.4%
Swim	8	0	**28**	0	82.4%	83.3%	77.8%	80%
V. Swim	0	0	0	**8**	100%	100%	100%	100%
RF (2 s epoch test)								
Chew	**10**	0	4	0	77%	94.2%	71.4%	74.1%
Rest	1	**21**	2	0	100%	95.1%	87.5%	93.3%
Swim	2	0	**34**	0	85%	95.2%	94.4%	89.5%
V. Swim	0	0	0	**8**	100%	100%	100%	100%
RF (1 s epoch test)								
Chew	**7**	2	5	0	70%	90.3%	50%	58.3%
Rest	1	**21**	2	0	91.3%	95%	87.5%	89.4%
Swim	2	0	**34**	0	83%	95.1%	94.4%	88.3%
V. Swim	0	0	0	**8**	100%	100%	100%	100%
XGB (2 s epoch test)								
Chew	**11**	0	3	0	68.8%	95.5%	78.6%	73.3%
Rest	1	**21**	2	0	100%	95.1%	87.5%	93.3%
Swim	4	0	**32**	0	86.5%	91.1%	88.9%	87.7%
V. Swim	0	0	0	**8**	100%	100%	100%	100%
XGB (1 s epoch test)								
Chew	**5**	1	8	0	55.6%	87.7%	35.7%	43.5%
Rest	0	**21**	3	0	91.3%	95%	87.5%	89.4%
Swim	4	1	**31**	0	73.8%	87.5%	86.1%	79.5%
V. Swim	0	0	0	**8**	100%	100%	100%	100%

## Supplementary Materials

The following are available online at https://www.mdpi.com/1424-8220/20/24/7096/s1. Table S1. Ethogram for behaviour labelling each discrete behaviour event used for modelling. Table S2. The numbers of behaviour events and data points per behaviour event for each of the four behaviour classes for 1 and 2 s epochs. Table S3. Results of Welch two sample t-test comparing behaviour categories: swimming on the floor and swimming in the water column. Table S4. Best parameters for all models created using 2 s sampling epoch. Figure S1. Boxplots for calculated metrics of accelerometry for behaviours: swimming on the floor (swimfloor) and swimming in the water column (swimcolumn). Figure S2. SVM, RF, and XGB 2 s model feature importance across four behaviour categories.
