# Supplementary material for: Assessment of Machine Learning Models to Identify Port Jackson Shark Behaviours Using Tri-Axial Accelerometers
Source: Sensors (Basel). 2020 Dec 11;20(24):7096. doi: 10.3390/s20247096 (PMC7763149; doi:10.3390/s20247096)
Supplement: Supplementary file 1 [file sensors-20-07096-s001.pdf]

Supplementary Information

Table S1. Ethogram for behaviour labelling each behaviour event used for modelling. Note, for modelling analysis swimming (floor) and swimming (column) were combined.

Behaviour	Description	Label begins	Label ends	Usually followed by (behaviour)
**Resting**	Immobile with both pectoral fins and tail on the floor	Begins when tail ceases motion and lower lobe of caudal fin first touches floor	Ends when tail first begins to undulate from straight resting position	Swimming, Chewing
**Swimming (floor)**	Mobile with one or more fins in contact with floor	Begins when tail first begins to undulate from straight resting position	Ends when movement stops and tail is no longer undulating	Resting, Swimming (column)
**Swimming (column)**	Mobile with no fins in contact with the floor	Begins when all fins leave the floor during swimming	Ends when one or more fins in contact with floor	Swimming (floor), Vertical Swimming
**Vertical Swimming**	Mobile in the water column with head upward	Begins when body is near perpendicular to the floor ≥ 45° angle	Ends when body is no longer at a ≥ 45° angle with floor	Swimming (column)
**Chewing**	Masticating/moving jaw up and down (does not include coughing behaviour)	Begins when prey item first touches jaw	Ends when last mastication is complete (i.e. jaw ceases movement)	Resting

Table S2. The numbers of behaviour events and data points per behaviour event for each of the four behaviour classes within the 1 and 2 second epochs.

	Epoch		
	**Behaviour events**	**Data points**	
**Behaviour**		**1 second**	**2 second**
**Resting**	122	1216	2407
**Swimming**	183	1823	3571
**Vertical swimming**	41	410	820
**Chewing**	74	734	1417
**Total**	420	4183	8215

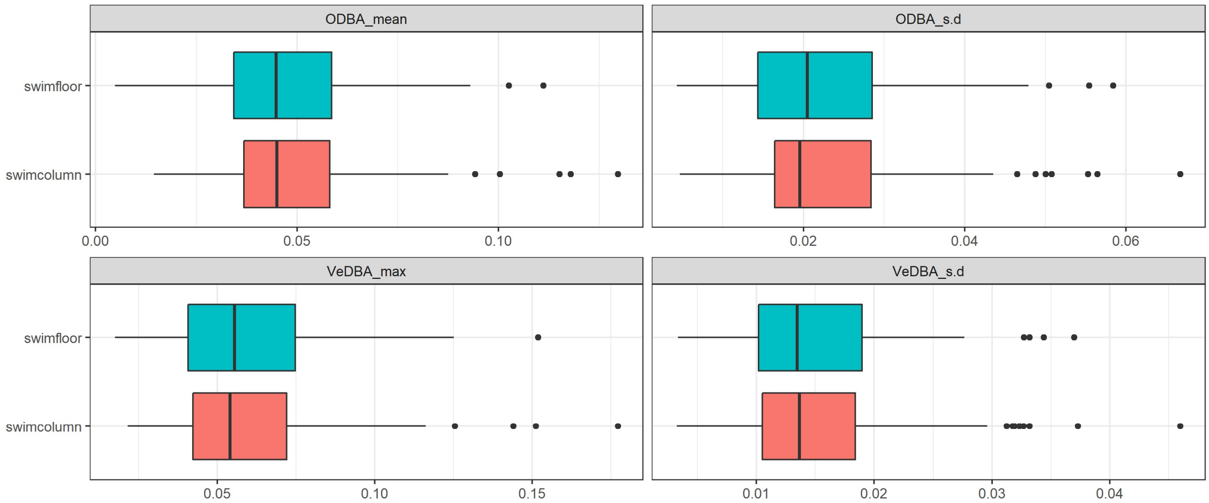

Figure S1. Boxplots for calculated metrics of accelerometry for behaviours: swimming on the floor (swimfloor) and swimming in the water column (swimcolumn). ODBA is overall dynamic body acceleration, VeDBA is vectorial dynamic body acceleration and s.d is standard deviation.

Table S3. Results of Welch two sample t-test comparing behaviour categories: swimming on the floor and swimming in the water column.

Acceleration metric	df	*p*	Swim column mean	Swim floor mean
ODBA mean	180	0.565	0.049	0.048
ODBA SD	181	0.689	0.023	0.023
VeDBA SD	179	0.576	0.016	0.015
VeDBA max	181	0.70	0.061	0.06

Table S4. Best parameters for all models created using 2 s sampling epoch.

Model	Best Parameters
**SVM**	Trials = 28; model = 2; winnow = FALSE
**RF**	mtry = 12
**XGB**	cp = 0.00929586494283577
**GBM**	size=5; decay=0.1; bag=FALSE
**C50**	sigma=0.0558887817581717; C=32
**CART**	n.trees=100; interaction.depth=2; shrinkage=0.05; n.minobsinnode=10
**Nnet**	nrounds=100; max_depth=1; eta=0.3; gamma=0; c olsample_bytree = 0.8; min_child_weight =1; subsample=0.5
**AvNnet**	size = 5; decay = 0.1

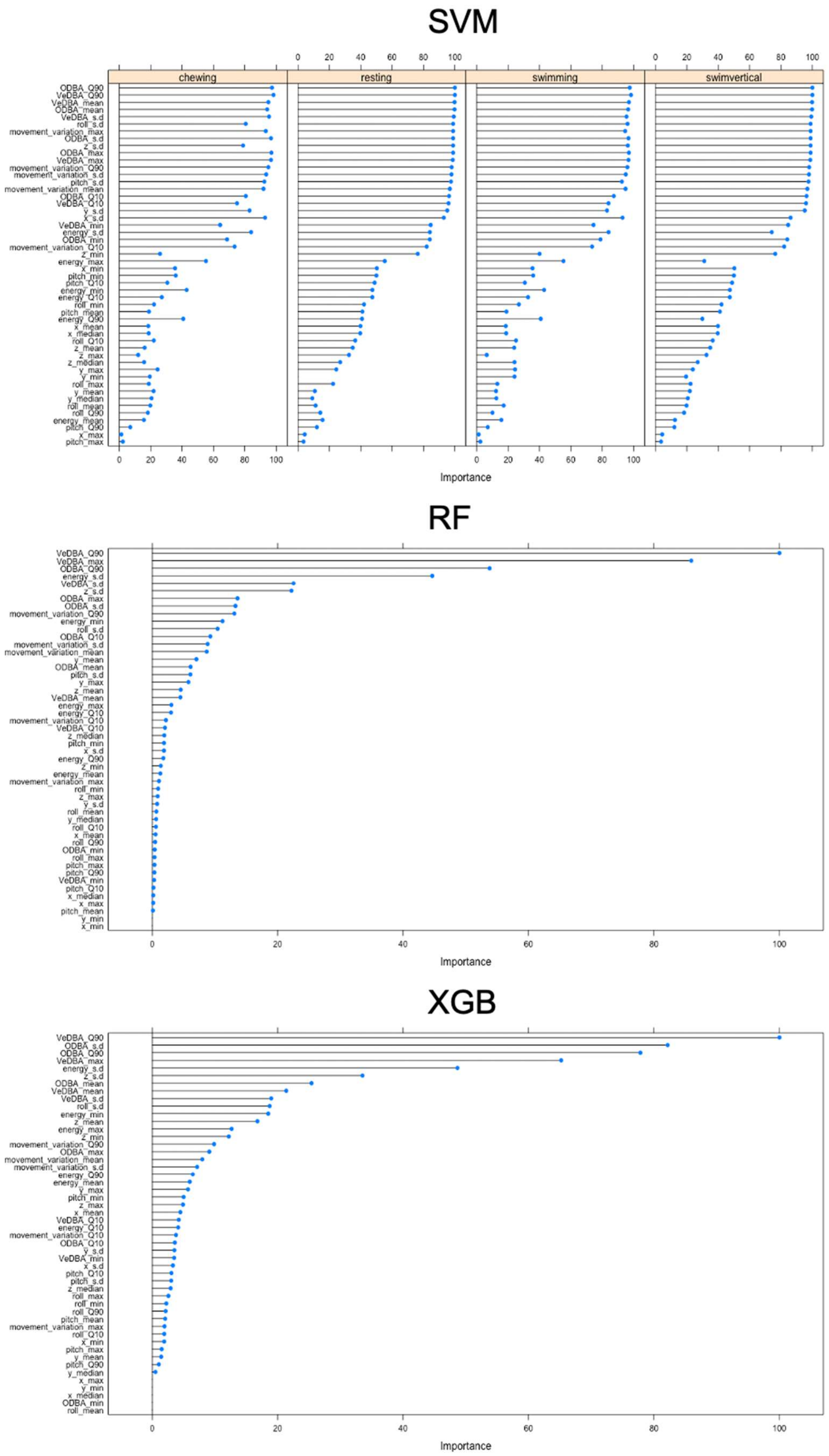

Figure S2. SVM, RF, and XGB 2 s model feature importance across four behaviour categories
